# Can mobile health apps replace GPs? A scoping review of comparisons between mobile apps and GP tasks

**DOI:** 10.1186/s12911-019-1016-4

**Published:** 2020-01-06

**Authors:** Apichai Wattanapisit, Chin Hai Teo, Sanhapan Wattanapisit, Emylia Teoh, Wing Jun Woo, Chirk Jenn Ng

**Affiliations:** 1 School of Medicine, Walailak University, Thasala, Nakhon Si Thammarat, Thailand; 2 Walailak University Hospital, Thasala, Nakhon Si Thammarat, Thailand; 3 Department of Primary Care Medicine, Faculty of Medicine, University of Malaya, Kuala Lumpur, Malaysia; 4 Thasala Hospital, Thasala, Nakhon Si Thammarat, Thailand; 5 Sechenov University, Moscow, Russia

**Keywords:** Clinical tasks, General practitioners, Mobile health applications

## Abstract

**Background:**

Mobile health applications (mHealth apps) are increasingly being used to perform tasks that are conventionally performed by general practitioners (GPs), such as those involved in promoting health, preventing disease, diagnosis, treatment, monitoring, and support for health services. This raises an important question: can mobile apps replace GPs? This study aimed to systematically search for and identify mobile apps that can perform GP tasks.

**Methods:**

A scoping review was carried out. The Google Play Store and Apple App Store were searched for mobile apps, using search terms derived from the UK Royal College of General Practitioners (RCGP) guideline on GPs’ core capabilities and competencies. A manual search was also performed to identify additional apps.

**Results:**

The final analysis included 17 apps from the Google Play Store and Apple App Store, and 21 apps identified by the manual search. mHealth apps were found to have the potential to replace GPs for tasks such as recording medical history and making diagnoses; performing some physical examinations; supporting clinical decision making and management; assisting in urgent, long-term, and disease-specific care; and health promotion. In contrast, mHealth apps were unable to perform medical procedures, appropriately utilise other professionals, and coordinate a team-based approach.

**Conclusions:**

This scoping review highlights the functions of mHealth apps that can potentially replace GP tasks. Future research should focus on assessing the performance and quality of mHealth apps in comparison with that of real doctors.

## Background

Mobile technology is rapidly transforming health care, education, and research [1, 2]. Globally, the number of smart phone subscriptions increased significantly from 1800 million in 2013 to 2600 million in 2014, and is estimated to reach 6100 million in 2020 [3]. Mobile health (mHealth) is defined as ‘medical and public health practice supported by mobile devices, such as mobile phones, patient-monitoring devices, personal digital assistants (PDAs), and other wireless devices’ [4]. The usage of mHealth has changed over time, since the first mobile phone was launched in the 1970s to the era of smart phones and wearable devices [5, 6]. mHealth has evolved beyond mobile devices to adopt diverse designs and perform a range of functions. Both patients and health professionals use mHealth for various reasons. According to a survey by the World Health Organization (WHO), mHealth is utilised for 1) communication between individuals and health services (e.g. health call centres); 2) communication between health services and individuals (e.g. appointment reminders); 3) consultation between health care professionals; 4) intersectoral communication in emergencies; 5) health monitoring and surveillance; and 6) access to information for health care professionals at the point of care [4]. The use of mobile devices in health care is considered to alter the delivery, quality, costs, and culture of health care [7, 8].

mHealth can reach large numbers of people and is not limited by borders; more than 100,000 health applications (apps) are available for mobile devices [9]. mHealth has grown dramatically and is beneficial for health care [10], performing numerous tasks such as diagnosing diseases, making appointments, keeping medical records, and supporting clinical decision-making [11–13]. However, the disadvantages of using mHealth in health care include the cost of technology and infrastructure, information security, lack of regulatory compliance guidelines, and the potential for serving as a workplace distraction [14, 15]. Moreover, accessibility to mHealth is a major concern in the context of equity in health care services. Evidence shows that the rate of mobile phone subscriptions among the population differs between countries worldwide. For example, in 2009, there were 202.99 subscriptions per 100 population in Estonia and 2.78 subscriptions per 100 population in Eritrea [4].

mHealth also plays important roles in primary care. A study conducted in the USA investigated the use of mobile devices and mHealth for health purposes among patients in primary care. The results showed that 90.1% of outpatients owned mobile phones, 55.3% of patients used smart phones, and 38.5% of patients (69.5% of smart phone owners) used mHealth [16]. Among all patients in this study, 35.5% sought health information from their smart phones, 22.0% accessed an mHealth app, and 20.8% tracked or managed health conditions via mobile devices [16]. Another study presented a new approach in which primary care practitioners prescribed mHealth apps to their patients and discussed the health data collected from the apps in subsequent patient visits [17]. A study of perceptions on mHealth in primary care in Belgium revealed that, among 111 adults from the general population, 41% used mHealth apps for general health check-ups, 18% for follow-up of chronic illnesses, 12% for post-hospitalisation monitoring, and 5% for tele-consultations instead of visiting doctors or hospitals [18]. Moreover, mHealth can contribute to the availability of more real-time and trended data instead of snapshots of the information based on serial visits [19].

Mobile apps are a vital component of mHealth [20, 21]. mHealth apps have been used in health promotion and disease prevention, diagnosis, treatment, monitoring, and the provision of support for health services [5]. These are typically clinical tasks that are conventionally performed by general practitioners (GPs) [22, 23]. Each mHealth app can perform a specific task or several tasks. Accordingly, patients may use a combination of mHealth apps on their mobile devices, which can help them to receive their needs, instead of seeing a GP. This raises the following important question: can mobile apps replace GPs? Although mHealth-related technologies are well designed and constructed, the functionality of these technologies are yet to be compared to the abilities of real doctors. This article aims to comprehensively review mobile apps that can perform GP tasks, and presents a comparison of the possible capabilities of such apps with those of real doctors.

## Methods

The authors conducted this scoping review following the PRISMA extension for scoping reviews (PRISMA-ScR) [24].

### Identifying GP tasks

This review used the Royal College of General Practitioners (RCGP) guideline on GPs’ core capabilities and competencies as a review framework [25]. In this context, a task is defined as an action relating to doctor-patient interaction performed by a GP during a clinical consultation. Two authors (AW and CHT) independently identified the tasks that should be performed by a GP based on the description of the RCGP guideline. Another author (CJN) participated in conflict resolution between the first two authors. This guideline comprises 13 capabilities and 31 competencies, with 12 tasks identified (Table 1).

**Table 1:** GPs’ core capabilities, competencies, and identified tasks

No.	Competency^a^	Is this a GP’s task?^b^	Search term
Fitness to practice			
1	Develop the attitudes and behaviours expected of a good doctor	No	N/A
2	Manage the factors that influence your performance	No	N/A
Maintaining and ethical approach			
3	Treat others fairly and with respect, acting without discrimination	No	N/A
4	Provide care with compassion and kindness	No	N/A
Communication and consultation			
5	Establish an effective partnership with patients	No	N/A
6	Maintain a continuing relationship with patients, carers and families	No	N/A
Data gathering and interpretation			
7	Apply a structured approach to data gathering and investigation	Yes	History taking
8	Interpret findings accurately to reach a diagnosis	Yes	Diagnosis
Clinical examinations and procedures			
9	Demonstrate a proficient approach to clinical examination	Yes	Clinical examination
10	Demonstrate a proficient approach to the performance of procedures	Yes	Medical procedures
Making decisions			
11	Adopt appropriate decision-making principles	Yes	Medical decision making
12	Apply a scientific and evidence-based approach	No	N/A
Clinical management			
13	Provide general clinical care to patients of all ages and backgrounds	No	N/A
14	Adopt a structured approach to clinical management	Yes	Clinical management
15	Make appropriate use of other professionals and services	Yes	Health professionals
16	Provide urgent care when needed	Yes	Urgent care
Managing medical complexity			
17	Enable people living with long-term conditions to improve their health	Yes	Long-term care
18	Manage concurrent health problems in an individual patient	Yes	Health problems
19	Adopt safe and effective approaches for patients with complex health needs	No	N/A
Working with colleagues and in teams			
20	Work as an effective team member	No	N/A
21	Coordinate a team-based approach to the care of patients	Yes	Team-based care
Maintaining performance learning and teaching			
22	Continuously evaluate and improve the care you provide	No	N/A
23	Adopt a safe and scientific approach to improve quality of care	No	N/A
24	Support the education and development of colleagues	No	N/A
Organisational management and leadership			
25	Apply leadership skills to help improve your organisation’s performance	No	N/A
26	Develop the financial and business skills required for your role	No	N/A
27	Make effective use of information management and communication systems	No	N/A
Practising holistically and promoting health			
28	Demonstrate the holistic mindset of a generalist medical practitioner	No	N/A
29	Support people through individual experiences of health, illness and recovery	Yes	Health promotion
Community orientation			
30	Understand the health service and your role within it	No	N/A
31	Build relationships with the communities with which you work	No	N/A

*N/A* not applicable

^a^GPs’ core capabilities and competencies based on the Royal College of General Practitioners (RCGP) guideline

^b^A task is defined as an action relating to doctor-patient interaction performed by a GP during a clinical consultation

### App search

Two authors (AW and SW) developed a search term for each task relating to doctor-patient interaction, and identified the final search terms via discussion with the rest of the authors. An author (AW) searched the Google Play Store and two authors (CHT and ET) searched the Apple App Store in July 2018, using the search term for each task. The authors used an Android device for searching Google Play Store and an iOS device for Apple App Store. The authors found some search terms yielded an uncountable list of apps, and most of them were irrelevant, especially the list after the first 20 apps. Thereafter, the author listed the first 20 apps for each task from each app database (Google Play Store and Apple App Store) for subsequent screening.

### Screening and selection of apps

Apps that can be used to perform clinical tasks and provide information in English were included. Those that were developed as electronic textbooks, training apps, and games were excluded. Up to the first 20 apps identified based on the Google Play Store and Apple App Store search results for each task, after the exclusion of duplicates and non-English apps, were considered eligible apps. Within each task, two independent review teams reviewed the apps independently: Team 1 (AW and SW) reviewed Android apps from the Google Play Store, while Team 2 (CHT and WJW) reviewed iOS apps from the Apple App Store. Each team assessed the relevant apps based on the app names and descriptions to determine whether they were capable of performing the relevant tasks. In case of uncertainty, the full apps were downloaded and assessed.

Apps that were found to perform several tasks were counted separately. The eligible mHealth apps were those that could perform specific tasks independently without the requirement to consult a real doctor. The final list of mHealth apps was identified after removing duplicates for each task. The authors summarised the results and resolved disagreements through consensus. According to a small number of apps included, the authors identified additional mHealth apps for Android (Google Play Store) and iOS (Apple App Store) mobile devices by using the search terms to search on web browsers manually. The relevant apps were selected by the consensus of the authors.

### Data charting process

The tasks were described using the search terms. The number of apps identified via a search of the Google Play Store and Apple App Store, as well as the total number, were presented. Additionally, the number of apps identified by the manual search was reported separately.

## Results

### Summary of app search results

**Fig. 1:**
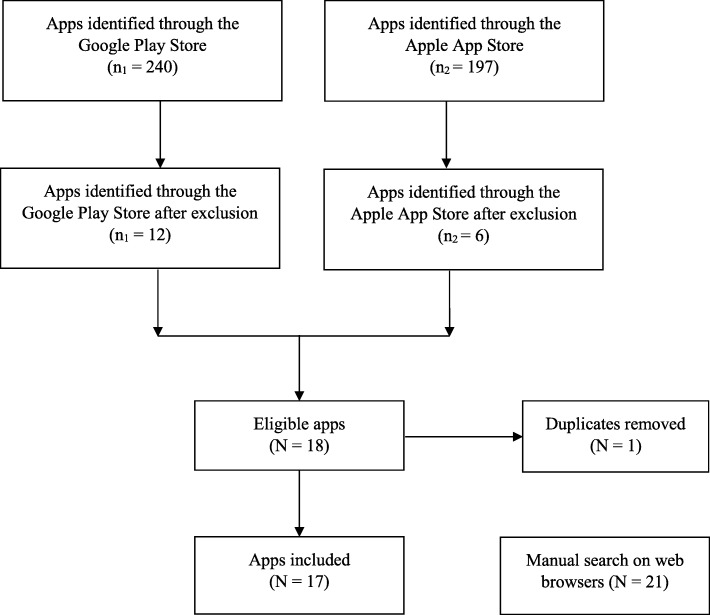
Flow diagram of the app review process

The initial search performed using the search terms for the 12 tasks revealed 437 apps from the Google Play Store (n_1_ = 240) and Apple App Store (n_2_ = 197). A total of 419 apps were excluded due to irrelevant app names, descriptions, and functions compared with the identified tasks by two independent review teams (Additional file 1). Of the 18 eligible apps, one duplicate within the same task was removed. The final analysis of apps from the Google Play Store and Apple App Store included 17 apps. The manual search on web browsers revealed an additional 21 apps. Figure 1 presents the app review flow diagram.

### Summary of the comparison between mobile apps and GP tasks

This scoping review revealed that nine out of 12 tasks could be potentially replaced by mHealth apps. The apps found by searching the Google Play Store and Apple App Store addressed the following three tasks: 1) apply a structured approach to data gathering and investigation; 2) interpret findings accurately to reach a diagnosis; and 3) support people through individual experiences of health, illness, and recovery. The manual search on web browsers identified several mHealth apps that were able to replace GPs in performing more clinical tasks (Table 2). A summary of the mHealth apps that can perform clinical tasks is presented in Table 3.

**Table 2:** Tasks of a GP that can be potentially performed by mHealth apps

No.	Task	Search term	Apps from the Google Play Store	Apps from the Apple App Store	Apps from the manual search
1	Apply a structured approach to data gathering and investigation	History taking	*N* = 2		n_3_ = 2 1. Ada 2. Babylon
			n_1_ = 1 1. Medical history builder	n_2_ = 1 1. Historian	
2	Interpret findings accurately to reach a diagnosis	Diagnosis	*N* = 9		n_3_ = 1 1. Babylon
			n_1_ = 7 1. Ada 2. Doctor Diagnose Symptoms Check 3. GBDiagnosis Medical App 4. My diagnostic 5. Self Diagnosis 6. Symptomate – Symptom checker 7. WebMD	n_2_ = 2 1. Rapid diagnosis- mental health 2. Your rapid diagnosis- STD	
3	Demonstrate a proficient approach to clinical examination	Clinical examination	*N* = 0		n_3_ = 2 1. Runtastic Heart Rate 2. SkinVision
			n_1_ = 0	n_2_ = 0	
4	Demonstrate a proficient approach to the performance of procedures	Medical procedures	*N* = 0		n_3_ = 0
			n_1_ = 0	n_2_ = 0	
5	Adopt appropriate decision-making principles	Medical decision making	*N* = 0		n_3_ = 1 1. Gout Decision Aid
			n_1_ = 0	n_2_ = 0	
6	Adopt a structured approach to clinical management	Clinical management	*N* = 0		n_3_ = 2 1. RapidDiagnosisMental Health 2. RapidDiagnosisSTD
			n_1_ = 0	n_2_ = 0	
7	Make appropriate use of other professionals and services	Health professionals	*N* = 0		n_3_ = 0
			n_1_ = 0	n_2_ = 0	
8	Provide urgent care when needed	Urgent care	*N* = 0		n_3_ = 2 1. Google Assistant 2. Siri
			n_1_ = 0	n_2_ = 0	
9	Enable people living with long-term conditions to improve their health	Long-term care	*N* = 0		n_3_ = 5 1. Asthma Manager 2. Blood Pressure Companion 3. mySugr 4. forDiabetes: diabetes self-management app 5. Pill Reminder and Medication Tracker by Medisafe
			n_1_ = 0	n_2_ = 0	
10	Manage concurrent health problems in an individual patient	Health problems	*N* = 0		n_3_ = 5 1. Asthma Manager 2. Blood Pressure Companion 3. mySugr 4. forDiabetes: diabetes self-management app 5. Pill Reminder and Medication Tracker by Medisafe
			n_1_ = 0	n_2_ = 0	
11	Coordinate a team-based approach to the care of patients	Team-based care	*N* = 0		n_3_ = 0
			n_1_ = 0	n_2_ = 0	
12	Support people through individual experiences of health, illness, and recovery	Health promotion	*N* = 6^a^		n_3_ = 1 1. BECCA - Breast Cancer Support
			n_1_ = 4 1. Appibuddy 2. Food (lg) 3. HealthHub Track 4. Healthy 365	n_2_ = 3 1. HealthWatch 2. Healthy 365 3. The circle of health	

*N* total number of apps, *n*_*1*_ number of apps from the Google Play Store, *n*_*2*_ number of apps from the Apple App Store, *n*_*3*_ number of apps from the manual search

^a^Total number after deduplication

**Table 3:** Summary of the apps

No.	App	Source	Objective of the app	Function of the app
Data gathering and interpretation Task: Apply a structured approach to data gathering and investigation				
1	Ada	Manual search	The app provides a personalised interactive chat to find possible explanations for illnesses.	The app can interview patients by using a series of questions, including those addressing the chief complaints and associated symptoms.
2	Babylon	Manual search	The app uses AI to analyse health problems and provide health advice.	The app collects patients’ information through a series of interactive questions.
3	Historian	Apple App Store	The app enables patients to enter a comprehensive psychiatric history and generate a detailed self-report of their mental state.	The app enables patients to evaluate their own mental health.
4	Medical history builder	Google Play Store	The app guides users in compiling their medical history in a systematic manner before seeing a doctor.	The app eliminates the need for a doctor to obtain a medical history from patients.
Data gathering and interpretation Task: Interpret findings accurately to reach a diagnosis				
5	Ada	Google Play Store	See no.1	The app produces a list of provisional and differential diagnoses based on the information collected by AI.
6	Babylon	Manual search	See no.2	The app enables the identification of possible causes of the symptoms entered by the user.
7	Doctor Diagnose Symptoms Check	Google Play Store	The app intends to inform and make patients more aware of their conditions.	The app can assist with symptom analysis and diagnosis.
8	GBDiagnosis Medical App	Google Play Store	The app identifies a suspected diagnosis based on the interaction and responses of users.	The app offers a simulated conversation between doctor and patient to collect symptoms and individual information to make a diagnosis.
9	My diagnostic	Google Play Store	The app aims to identify diseases in a database based on the symptoms entered by users.	The app requires user information regarding their symptoms to make a diagnosis.
10	Rapid diagnosis- mental health	Apple App Store	The app is designed to assist with the diagnosis of mental, emotional, or psychological conditions that can be differentiated based on symptoms.	The app is used as a symptom checker, and a probable diagnosis can be established.
11	Rapid diagnosis- STD	Apple App Store	The app is designed to assist with the diagnosis of sexually transmitted diseases.	The app is used as a symptom checker, and allows a probable diagnosis to be established.
12	Self Diagnosis	Google Play Store	The app enables the user to identify their condition	The app can make a diagnosis based on the responses of the user.
13	Symptomate – Symptom checker	Google Play Store	The app provides an evaluation of users’ health.	The app indicates possible causes of symptoms, treatment options, and suggested lab tests.
14	WebMD	Google Play Store	The app offers doctor-reviewed health information and interactive tools.	The app includes the function ‘Symptom Checker’ and provides a list of possible diagnoses based on a major symptom and a brief set of general questions (current medications and current and past illnesses).
Clinical examinations and procedures Task: Demonstrate a proficient approach to clinical examination				
15	Runtastic Heart Rate	Manual search	The app is used for checking heart rate anytime and anywhere.	The app measures heart rate by tapping a finger on the smart phone camera.
16	SkinVision	Manual search	The app checks the skin for signs of skin cancer.	The app uses the phone camera to capture an image of a skin lesion and evaluate the user’s risk of skin cancer.
Making decisions Task: Adopt appropriate decision-making principles				
17	Gout Decision Aid	Manual search	The app provides information and education to patients with gout.	The app can function as a patient decision aid and a tool to help the user becomes involved in decision making.
Clinical management Task: Adopt a structured approach to clinical management				
18	Rapid diagnosis- mental health	Manual search	See no.10	The app offers information on patient management related to the diagnosis of mental illnesses.
19	Rapid diagnosis- STD	Manual search	See no.11	The app offers information on patient management related to the diagnosis of sexually transmitted diseases.
Clinical management Task: Provide urgent care when needed				
20	Google Assistant	Manual search	The app provides AI to assist users in utilising phone functions and searching for information on the internet.	The app can provide information on urgent and emergency conditions and can navigate users to the nearest hospital.
21	Siri	Manual search	The app runs on the iOS platform (Apple devices); its AI functions as a virtual assistant.	The app can provide information on urgent and emergency conditions and can navigate users to the nearest hospital.
Managing medical complexity Task: Enable people living with long-term conditions to improve their health				
22	Asthma Manager	Manual search	The app is used to manage asthma.	The app can help track symptoms and manage medications.
23	Blood Pressure Companion	Manual search	The app is designed for monitoring blood pressure.	The app can record and analyse blood pressure as well as generate graphs, charts, and tables of the results.
24	mySugr	Manual search	The app is a diabetes logbook for type 1, type 2, and gestational diabetes mellitus.	The app can record and analyse diabetes parameters as well as provide feedback.
25	forDiabetes: diabetes self-management app	Manual search	The app is designed for managing diabetes.	The app can track and monitor key diabetes data, including blood glucose level, HbA1c, blood pressure, and medications.
26	Pill Reminder and Medication Tracker by Medisafe	Manual search	The app is used as a medication reminder and enables medication tracking.	The app reminds users to take medications and produces daily and monthly medication progress reports.
Managing medical complexity Task: Manage concurrent health problems in an individual patient				
27	Asthma Manager	Manual search	See no.22–26	See no.22–26
28	Blood Pressure Companion	Manual search		
29	mySugr	Manual search		
30	forDiabetes: diabetes self-management app	Manual search		
31	Pill Reminder and Medication Tracker by Medisafe	Manual search		
Practising holistically and promoting health Task: Support people through individual experiences of health, illness and recovery				
32	Appibuddy	Google Play Store	The app is a healthy lifestyle platform focusing on weight reduction.	The app enables users to record health behaviours and provides a platform on which to share their activities and learn from other users.
33	BECCA - Breast Cancer Support	Manual search	The app aims to support and help users in living well after breast cancer.	The app provides health tips, information and blogposts to support patients in moving forward after cancer treatment.
34	Food (lg)	Google Play Store	The app is a food journal and nutrition tracker and analyser.	The app can analyse diet and calories by simply taking pictures of food.
35	HealthHub Track	Google Play Store	The app aims to achieve personalised health goals through tools, action plans, and healthy lifestyle guides.	The app can record health behaviours and provide personalised action plans based on the user’s goal.
36	HealthWatch	Apple App Store	The app aims to provide practical tools to maintain and enhance health and quality of life and counteract stress-related illnesses.	The app provides patient education and comprises a tool that records stress levels and provides feedback accordingly.
37	Healthy 365	Google Play Store and Apple App Store	The app promotes healthy lifestyles.	The app enables users to keep track of daily steps and calculate the number of calories burned.
38	The circle of health	Apple App Store	The app aims to promote cardiovascular health.	The app can assess and measure cardiovascular health and motivate users to maintain healthy habits.

*AI* artificial intelligence

*HbA1c* haemoglobin A1c

## Discussion

This scoping review identified mobile apps that are capable of performing GP tasks. mHealth apps were found to exhibit the potential to replace GPs in taking medical history and making a diagnosis; performing some physical examinations; supporting clinical decision-making and management; assisting in urgent, long-term, and disease-specific care; and performing health promotion. However, mHealth apps were unable to perform medical procedures, appropriately utilise other professionals, and coordinate a team-based approach.

mHealth apps serve diverse purposes and perform a range of functions for both patients and health care providers [26, 27]. This scoping review focused on mHealth apps utilised by patients for health purposes. The findings suggest that mHealth apps have the potential to perform several specific clinical tasks that are conventionally performed by a GP. Previous studies have reported the roles of mHealth apps for patients with specific clinical goals, such as pain self-management and weight management [28, 29]. Such mobile app functions may replace several GP tasks, for example, an app for diagnosis could help users make decisions regarding further treatment options, therefore potentially replacing a GP for this purpose. However, most apps, especially apps for history taking and diagnosis, have been found to lack the potential to replace a consultation with a GP. The apps were only found to be suitable for providing primary information and health-related suggestions.

Some GP tasks could not be performed by mHealth. For example, mHealth apps could not perform medical procedures. However, mHealth apps, together with other supportive technologies, have the potential to support clinical tasks. Examples of technologies capable of supporting mHealth approaches include near-field communication (NFC) (a short-range, wireless connectivity technology), accelerometers (a technology used to measure gravitational forces and accelerations), gyroscopes (a micro-electromechanical system sensor used to measure body movement), artificial intelligence (AI), and machine learning [30–32]. For example, NFC can be used to monitor human’s physiological information (e.g. heart rate, body temperature) [33]. Accelerometers and gyroscopes can function as motion sensors to monitor daily activities, falls, and sleep patterns [34].

The other competencies of GPs comprise personal attributes, including attitudes, practical skills, and soft skills, which mHealth apps cannot currently replace. Nevertheless, mHealth apps may support GPs in terms of training and referencing. In the future, the development of technologies could contribute to more efficient functions of the mHealth. For example, AI and machine learning may enable machines to learn essential skills, as well as develop attitudes and a mindset similar to those of a good doctor.

Although the findings revealed that mHealth apps were able to perform some GP tasks, it could not be concluded that mHealth apps could replace GPs. Being a medical doctor requires integrative skills, art, values, and ethics [35, 36]. For example, taking history without physical examination may lead to unnecessary investigations and a misdiagnosis. From the results of this review, some apps were able to perform multiple tasks. Their integrative functionality could not replace the comprehensive functions of GPs. Using modern technologies such as mHealth can facilitate the quality of care. Many mHealth apps offer platforms for telemedicine to facilitate doctor-patient communication, which is cost-effective and timely [37]. A study explored doctor-patient communication through screen-to-screen versus face-to-face consultations showed no significantly different results regarding the quality of doctor-patient communication [38]. However, using mHealth apps without human interactions cannot replace seeing a GP.

mHealth apps may additionally present several risks to the user, including loss of privacy, poor-quality patient data, and inappropriate clinical management of the user [39]. To address these risks, basic standards should be met, including accessibility, appropriate privacy, accuracy and credibility of content, and ethical obligations [40, 41]. The differing views in regard to medical technology among patients and doctors are also an area of concern [42]. Such differing perceptions may lead to misunderstandings and arguments between patients and doctors in general practice. GPs should aim to serve as expert sources of digital health information for their patients [43]. Therefore, ‘expertise in the use of appropriate mHealth-related technologies’ should be recognised as an additional competency of GPs.

The present scoping review was conducted based on the UK RCGP guideline as a framework. This approach enabled specification of the functions of mHealth apps compared with GP tasks, which was a major strength of this study. However, there were three limitations of this review. First, the search terms used may have limited the search results. This review used only one search term for each task and did not use any alternative terms. Second, the review did not include all mHealth apps from the Google Play Store and Apple App Store because an exhaustive list of all apps for some tasks was not possible to obtain. The authors resolved these problems by including only the first 20 apps found for each task from each app database. Additionally, to identify additional apps for each task, the authors performed a manual search based on discussion. Finally, this scoping review focused on the functions of apps, however, it was unable to evaluate the quality and credibility of the apps. This reflected a characteristic of scoping review, which primarily focused on identifying knowledge gaps and key characteristics related to a concept [44].

## Conclusions

mHealth apps have the potential to replace some GP tasks (nine out of 12 tasks), whereas a GP is expected to be competent in all tasks and with respect to all attributes. Innovative technologies, such as AI and machine learning, are anticipated to play important roles in improving mHealth apps to achieve the capability to perform additional GP tasks and possess more of their attributes. There is a need to balance the advantages and disadvantages of the use of mHealth in health care. GPs should understand and prevent the risks of using mHealth apps. Expertise in the use of appropriate mHealth-related technologies should be recognised as an essential competency of GPs. Future research should focus on assessing the performance and capabilities of mHealth apps compared with those of real doctors.

## Supplementary information

**Additional file 1.** Summary of screening and selection of apps.

## Data Availability

All data analysed during this study are included in this published article and its additional files.

## Abbreviations

AI: Artificial intelligence
Apps: Applications
GPs: General practitioners
mHealth: Mobile health
NFC: Near-field communication
PDAs: Personal digital assistants
RCGP: Royal College of General Practitioners
UK: United Kingdom
USA: United States of America
WHO: World Health Organization
