# Supplementary material for: Can mobile health apps replace GPs? A scoping review of comparisons between mobile apps and GP tasks
Source: BMC Med Inform Decis Mak. 2020 Jan 6;20:5. doi: 10.1186/s12911-019-1016-4 (PMC6945711; doi:10.1186/s12911-019-1016-4)
Supplement: Supplementary file 1 — Additional file 1. Summary of screening and selection of apps. [file 12911_2019_1016_MOESM1_ESM.docx]

**Additional File 1** Summary of screening and selection of apps

**Android apps (Team 1)**

| **1 Apply a structured approach to data gathering and investigation**  **Search term: History taking** | | | | | | | | | | | | | |
| --- | --- | --- | --- | --- | --- | --- | --- | --- | --- | --- | --- | --- | --- |
| **No** | | **App** | | **AW**  **(Relevant or Irrelevant)** | **SW**  **(Relevant or Irrelevant)** | | | | | | | **Consensus** | |
| 1 | | History Taking | | Irrelevant | Irrelevant | | | | | | | Irrelevant | |
| 2 | | History Clinic | | Irrelevant | Irrelevant | | | | | | | Irrelevant | |
| 3 | | Clinicals – History & Exam | | Irrelevant | Irrelevant | | | | | | | Irrelevant | |
| 4 | | Jaffy Skills | | Irrelevant | Irrelevant | | | | | | | Irrelevant | |
| 5 | | Medical history builder | | Relevant | Relevant | | | | | | | Relevant | |
| 6 | | Physical Examination & History Taking 8e | | Irrelevant | Irrelevant | | | | | | | Irrelevant | |
| 7 | | Bates' Physical Examination | | Irrelevant | Irrelevant | | | | | | | Irrelevant | |
| 8 | | Patient History Taker | | Irrelevant | Irrelevant | | | | | | | Irrelevant | |
| 9 | | MedEx - Clinical Examination | | Irrelevant | Irrelevant | | | | | | | Irrelevant | |
| 10 | | Clinical Examination Tips | | Irrelevant | Irrelevant | | | | | | | Irrelevant | |
| 11 | | Medical History | | Irrelevant | Irrelevant | | | | | | | Irrelevant | |
| 12 | | Clinical Pediatrics | | Irrelevant | Irrelevant | | | | | | | Irrelevant | |
| 13 | | Clinical History | | Irrelevant | Irrelevant | | | | | | | Irrelevant | |
| 14 | | Clinical Examination Mnemonics | | Irrelevant | Irrelevant | | | | | | | Irrelevant | |
| 15 | | Clinical Surgery | | Irrelevant | Irrelevant | | | | | | | Irrelevant | |
| 16 | | Common Symptom Guide | | Irrelevant | Irrelevant | | | | | | | Irrelevant | |
| 17 | | Geeky Medics - OSCE revision | | Irrelevant | Irrelevant | | | | | | | Irrelevant | |
| 18 | | Veena O&G | | Irrelevant | Irrelevant | | | | | | | Irrelevant | |
| 19 | | OSCEr | | Irrelevant | Irrelevant | | | | | | | Irrelevant | |
| 20 | | 100 Cases in Obstetrics and Gynaecology | | Irrelevant | Irrelevant | | | | | | | Irrelevant | |
| **2 Interpret findings accurately to reach a diagnosis**  **Search term: Diagnosis** | | | | | | | | | | | | | |
| **No** | | **App** | | **AW**  **(Relevant or Irrelevant)** | | **SW**  **(Relevant or Irrelevant)** | | | | | **Consensus** | | |
| 1 | | Prognosis : Your Diagnosis | | Irrelevant | | Irrelevant | | | | | Irrelevant | | |
| 2 | | 🇬🇧Diagnosis Medical App | | Relevant | | Relevant | | | | | Relevant | | |
| 3 | | Ada - Your Health Guide | | Relevant | | Relevant | | | | | Relevant | | |
| 4 | | Quick Medical Diagnosis & Treatment | | Irrelevant | | Irrelevant | | | | | Irrelevant | | |
| 5 | | Doctor Diagnose Symptoms Check | | Relevant | | Relevant | | | | | Relevant | | |
| 6 | | LG AC Smart Diagnosis | | Irrelevant | | Irrelevant | | | | | Irrelevant | | |
| 7 | | CAR DIAGNOSIS AND REPAIRS | | Irrelevant | | Irrelevant | | | | | Irrelevant | | |
| 8 | | disease diagnosis | | Irrelevant | | Irrelevant | | | | | Irrelevant | | |
| 9 | | History Clinic | | Irrelevant | | Irrelevant | | | | | Irrelevant | | |
| 10 | | Clinical Cases Diagnosis | | Irrelevant | | Irrelevant | | | | | Irrelevant | | |
| 11 | | Clinical Medicine Differential Diagnosis | | Irrelevant | | Irrelevant | | | | | Irrelevant | | |
| 12 | | Symptomate – Symptom checker | | Relevant | | Relevant | | | | | Relevant | | |
| 13 | | Cardiac diagnosis (heart rate, arrhythmia) | | Irrelevant | | Irrelevant | | | | | Irrelevant | | |
| 14 | | Symptom to Diagnosis | | Irrelevant | | Irrelevant | | | | | Irrelevant | | |
| 15 | | WebMD | | Relevant | | Relevant | | | | | Relevant | | |
| 16 | | My diagnostic | | Relevant | | Relevant | | | | | Relevant | | |
| 17 | | Nursing Diagnosis Flashcards 2018 | | Irrelevant | | Irrelevant | | | | | Irrelevant | | |
| 18 | | Self Diagnosis | | Relevant | | Relevant | | | | | Relevant | | |
| 19 | | Eye Diagnosis | | Irrelevant | | Irrelevant | | | | | Irrelevant | | |
| 20 | | DSM-5 Differential Diagnosis | | Irrelevant | | Relevant | | | | | Irrelevant | | |
| **3 Demonstrate a proficient approach to clinical examination**  **Search term: Clinical examination** | | | | | | | | | | | | | |
| **No** | | **App** | | **AW**  **(Relevant or Irrelevant)** | | | | **SW**  **(Relevant or Irrelevant)** | | | | **Consensus** | |
| 1 | | MedEx - Clinical Examination | | Irrelevant | | | | Irrelevant | | | | Irrelevant | |
| 2 | | Clinical Examination & Skill | | Irrelevant | | | | Irrelevant | | | | Irrelevant | |
| 3 | | Clinical Examination Mnemonics | | Irrelevant | | | | Irrelevant | | | | Irrelevant | |
| 4 | | Clinical Examination Tips | | Irrelevant | | | | Irrelevant | | | | Irrelevant | |
| 5 | | MedEx-Clinical Examination(pro) | | Irrelevant | | | | Irrelevant | | | | Irrelevant | |
| 6 | | Clinical Examination & Skills | | Irrelevant | | | | Irrelevant | | | | Irrelevant | |
| 7 | | Clinical Examinations for Students of Ayurveda | | Irrelevant | | | | Irrelevant | | | | Irrelevant | |
| 8 | | OSCE Reference Guide | | Irrelevant | | | | Irrelevant | | | | Irrelevant | |
| 9 | | Bates' Physical Examination | | Irrelevant | | | | Irrelevant | | | | Irrelevant | |
| 10 | | Pocket PEx | | Irrelevant | | | | Irrelevant | | | | Irrelevant | |
| 11 | | OSCEr | | Irrelevant | | | | Irrelevant | | | | Irrelevant | |
| 12 | | Common Symptom Guide | | Irrelevant | | | | Irrelevant | | | | Irrelevant | |
| 13 | | Pocket Tutor: Paediatric Clinical Examination | | Irrelevant | | | | Irrelevant | | | | Irrelevant | |
| 14 | | History Clinic | | Irrelevant | | | | Irrelevant | | | | Irrelevant | |
| 15 | | Physical Exam Essentials | | Irrelevant | | | | Irrelevant | | | | Irrelevant | |
| 16 | | Physical Examination & History Taking 8e | | Irrelevant | | | | Irrelevant | | | | Irrelevant | |
| 17 | | Examination of the heart | | Irrelevant | | | | Irrelevant | | | | Irrelevant | |
| 18 | | Medical examination | | Irrelevant | | | | Irrelevant | | | | Irrelevant | |
| 19 | | History Taking | | Irrelevant | | | | Irrelevant | | | | Irrelevant | |
| 20 | | Smart Medical Apps | | Irrelevant | | | | Irrelevant | | | | Irrelevant | |
| **4 Demonstrate a proficient approach to the performance of procedures**  **Search term: Medical procedures** | | | | | | | | | | | | | |
| **No** | | **App** | | **AW**  **(Relevant or Irrelevant)** | | | **SW**  **(Relevant or Irrelevant)** | | | | | **Consensus** | |
| 1 | | Medical & Surgical Procedure | | Irrelevant | | | Irrelevant | | | | | Irrelevant | |
| 2 | | Surgical & Medical Procedures | | Irrelevant | | | Irrelevant | | | | | Irrelevant | |
| 3 | | Internal Medicine Surgery Procedures | | Irrelevant | | | Irrelevant | | | | | Irrelevant | |
| 4 | | Basic Surgery | | Irrelevant | | | Irrelevant | | | | | Irrelevant | |
| 5 | | WikiMed - Offline Medical Wikipedia | | Irrelevant | | | Irrelevant | | | | | Irrelevant | |
| 6 | | Touch Surgery | | Irrelevant | | | Irrelevant | | | | | Irrelevant | |
| 7 | | Medical Surgical RN Companion | | Irrelevant | | | Irrelevant | | | | | Irrelevant | |
| 8 | | Lippincott Procedures | | Irrelevant | | | Irrelevant | | | | | Irrelevant | |
| 9 | | Nursing Procedures | | Irrelevant | | | Irrelevant | | | | | Irrelevant | |
| 10 | | thumbroll | | Irrelevant | | | Irrelevant | | | | | Irrelevant | |
| 11 | | Surgery Dictionary | | Irrelevant | | | Irrelevant | | | | | Irrelevant | |
| 12 | | MSD Manual Pro Version | | Irrelevant | | | Irrelevant | | | | | Irrelevant | |
| 13 | | Clinical Lab Sciences | | Irrelevant | | | Irrelevant | | | | | Irrelevant | |
| 14 | | All Stomach Diseases and Treatment | | Irrelevant | | | Irrelevant | | | | | Irrelevant | |
| 15 | | Clinical Skills | | Irrelevant | | | Irrelevant | | | | | Irrelevant | |
| 16 | | Learn Medical Instruments List | | Irrelevant | | | Irrelevant | | | | | Irrelevant | |
| 17 | | Clinical Treatment - Medical Diseases Treatment | | Irrelevant | | | Irrelevant | | | | | Irrelevant | |
| 18 | | Quick Medical Diagnosis & Treatment | | Irrelevant | | | Irrelevant | | | | | Irrelevant | |
| 19 | | Medical Instruments Basics | | Irrelevant | | | Irrelevant | | | | | Irrelevant | |
| 20 | | Procedures in O&G | | Irrelevant | | | Irrelevant | | | | | Irrelevant | |
| **5 Adopt appropriate decision-making principles**  **Search term: Medical decision making** | | | | | | | | | | | | | |
| **No** | | **App** | | **AW**  **(Relevant or Irrelevant)** | | | **SW**  **(Relevant or Irrelevant)** | | | | | **Consensus** | |
| 1 | | Clinical Decision Making | | Irrelevant | | | Irrelevant | | | | | Irrelevant | |
| 2 | | MDCalc Medical Calculator | | Irrelevant | | | Irrelevant | | | | | Irrelevant | |
| 3 | | Prognosis : Your Diagnosis | | Irrelevant | | | Irrelevant | | | | | Irrelevant | |
| 4 | | DecidApp Free. decision making | | Irrelevant | | | Irrelevant | | | | | Irrelevant | |
| 5 | | Medical News & Journals | | Irrelevant | | | Irrelevant | | | | | Irrelevant | |
| 6 | | ClinicalLaboratoryDiagnostics | | Irrelevant | | | Irrelevant | | | | | Irrelevant | |
| 7 | | The Chief Complaint | | Irrelevant | | | Irrelevant | | | | | Irrelevant | |
| 8 | | Resus Days | | Irrelevant | | | Irrelevant | | | | | Irrelevant | |
| 9 | | Medical Calculator & Equations | | Irrelevant | | | Irrelevant | | | | | Irrelevant | |
| 10 | | NCCN Guidelines for Smartphone | | Irrelevant | | | Irrelevant | | | | | Irrelevant | |
| 11 | | OrthoGuidelines | | Irrelevant | | | Irrelevant | | | | | Irrelevant | |
| 12 | | Medialis | | Irrelevant | | | Irrelevant | | | | | Irrelevant | |
| 13 | | MIMS Thailand - Drug Information, Disease, News | | Irrelevant | | | Irrelevant | | | | | Irrelevant | |
| 14 | | ACCA Toolkit | | Irrelevant | | | Irrelevant | | | | | Irrelevant | |
| 15 | | DIP | | Irrelevant | | | Irrelevant | | | | | Irrelevant | |
| 16 | | Urticaria Xplained | | Irrelevant | | | Irrelevant | | | | | Irrelevant | |
| 17 | | SMDM | | Irrelevant | | | Irrelevant | | | | | Irrelevant | |
| 18 | | MedEdCases | | Irrelevant | | | Irrelevant | | | | | Irrelevant | |
| 19 | | 100 Cases in Obstetrics and Gynaecology | | Irrelevant | | | Irrelevant | | | | | Irrelevant | |
| 20 | | Probiotic Guide US | | Irrelevant | | | Irrelevant | | | | | Irrelevant | |
| **6** **Adopt a structured approach to clinical management**  **Search term: Clinical management** | | | | | | | | | | | | | |
| **No** | | **App** | | **AW**  **(Relevant or Irrelevant)** | | | | **SW**  **(Relevant or Irrelevant)** | | | | **Consensus** | |
| 1 | | MedDNA - Clinic Management | | Irrelevant | | | | Irrelevant | | | | Irrelevant | |
| 2 | | Clinical Treatment - Medical Diseases Treatment | | Irrelevant | | | | Irrelevant | | | | Irrelevant | |
| 3 | | My Clinic Hospital-Patient Mgt | | Irrelevant | | | | Irrelevant | | | | Irrelevant | |
| 4 | | HSM - Clinic Management System | | Irrelevant | | | | Irrelevant | | | | Irrelevant | |
| 5 | | Patient Medical Records & Appointments for Doctors | | Irrelevant | | | | Irrelevant | | | | Irrelevant | |
| 6 | | MyOPD™ - Practice Management | | Irrelevant | | | | Irrelevant | | | | Irrelevant | |
| 7 | | Symptom Management Guides | | Irrelevant | | | | Irrelevant | | | | Irrelevant | |
| 8 | | Appointik - Medical Practice Management | | Irrelevant | | | | Irrelevant | | | | Irrelevant | |
| 9 | | Smarte Clinic GTB Patient Management System | | Irrelevant | | | | Irrelevant | | | | Irrelevant | |
| 10 | | Clinical Sense | | Irrelevant | | | | Irrelevant | | | | Irrelevant | |
| 11 | | Medical Records App | | Irrelevant | | | | Irrelevant | | | | Irrelevant | |
| 12 | | Quick Medical Diagnosis & Treatment | | Irrelevant | | | | Irrelevant | | | | Irrelevant | |
| 13 | | The Chief Complaint | | Irrelevant | | | | Irrelevant | | | | Irrelevant | |
| 14 | | Doctor Patient Diary | | Irrelevant | | | | Irrelevant | | | | Irrelevant | |
| 15 | | Medical Management of MDR-TB | | Irrelevant | | | | Irrelevant | | | | Irrelevant | |
| 16 | | All Stomach Diseases and Treatment | | Irrelevant | | | | Irrelevant | | | | Irrelevant | |
| 17 | | AugmentCare for Doctors: Manage Your Practice | | Irrelevant | | | | Irrelevant | | | | Irrelevant | |
| 18 | | Diseases Dictionary ✪ Medical | | Irrelevant | | | | Irrelevant | | | | Irrelevant | |
| 19 | | SmartClinic | | Irrelevant | | | | Irrelevant | | | | Irrelevant | |
| 20 | | Clinical Practice Guidelines | | Irrelevant | | | | Irrelevant | | | | Irrelevant | |
| **7 Make appropriate use of other professionals and services**  **Search term: Health professionals** | | | | | | | | | | | | | |
| **No** | | **App** | | **AW**  **(Relevant or Irrelevant)** | | | **SW**  **(Relevant or Irrelevant)** | | | | | **Consensus** | |
| 1 | | MyDoc Pro Health Professionals | | Irrelevant | | | Irrelevant | | | | | Irrelevant | |
| 2 | | Medical Professionals | | Irrelevant | | | Irrelevant | | | | | Irrelevant | |
| 3 | | Health Professions and Nursing | | Irrelevant | | | Irrelevant | | | | | Irrelevant | |
| 4 | | San Xavier Health Professionals | | Irrelevant | | | Irrelevant | | | | | Irrelevant | |
| 5 | | Mosby's Drug Reference for Health Professions | | Irrelevant | | | Irrelevant | | | | | Irrelevant | |
| 6 | | Health Care Professionals Apps | | Irrelevant | | | Irrelevant | | | | | Irrelevant | |
| 7 | | Health Jobs | | Irrelevant | | | Irrelevant | | | | | Irrelevant | |
| 8 | | GoConsultPro - Health | | Irrelevant | | | Irrelevant | | | | | Irrelevant | |
| 9 | | Virtual Practice for Healthcare Providers | | Irrelevant | | | Irrelevant | | | | | Irrelevant | |
| 10 | | Private Health Professionals | | Irrelevant | | | Irrelevant | | | | | Irrelevant | |
| 11 | | HCP Space | | Irrelevant | | | Irrelevant | | | | | Irrelevant | |
| 12 | | Medscape | | Irrelevant | | | Irrelevant | | | | | Irrelevant | |
| 13 | | MaNaDr for Healthcare Provider | | Irrelevant | | | Irrelevant | | | | | Irrelevant | |
| 14 | | NexJ Health Coach | | Irrelevant | | | Irrelevant | | | | | Irrelevant | |
| 15 | | Bhalo Achi \| Into healthcare | | Irrelevant | | | Irrelevant | | | | | Irrelevant | |
| 16 | | Azova | | Irrelevant | | | Irrelevant | | | | | Irrelevant | |
| 17 | | Skyscape Medical Library | | Irrelevant | | | Irrelevant | | | | | Irrelevant | |
| 18 | | Doctoralia | | Irrelevant | | | Irrelevant | | | | | Irrelevant | |
| 19 | | World Medical Card | | Irrelevant | | | Irrelevant | | | | | Irrelevant | |
| 20 | | Epocrates | | Irrelevant | | | Irrelevant | | | | | Irrelevant | |
| **8 Provide urgent care when needed**  **Search term: Urgent care** | | | | | | | | | | | | | |
| **No** | | **App** | | **AW**  **(Relevant or Irrelevant)** | | | | **SW**  **(Relevant or Irrelevant)** | | | | **Consensus** | |
| 1 | | Urgent Care (ChARM Health Apps/MedicalMine) | | Irrelevant | | | | Irrelevant | | | | Irrelevant | |
| 2 | | Urgent Care RAP | | Irrelevant | | | | Irrelevant | | | | Irrelevant | |
| 3 | | Urgent Care (onlinecare) | | Irrelevant | | | | Irrelevant | | | | Irrelevant | |
| 4 | | Urgent Care & Hospital Locator | | Irrelevant | | | | Irrelevant | | | | Irrelevant | |
| 5 | | Ambulatory and Urgent Care | | Irrelevant | | | | Irrelevant | | | | Irrelevant | |
| 6 | | FaceCure Virtual Urgent Care | | Irrelevant | | | | Irrelevant | | | | Irrelevant | |
| 7 | | XpediCare online urgent care | | Irrelevant | | | | Irrelevant | | | | Irrelevant | |
| 8 | | palmEM: Emergency Medicine | | Irrelevant | | | | Irrelevant | | | | Irrelevant | |
| 9 | | WikEM - Emergency Medicine | | Irrelevant | | | | Irrelevant | | | | Irrelevant | |
| 10 | | D2DUC - Door to Door Urgent Care | | Irrelevant | | | | Irrelevant | | | | Irrelevant | |
| 11 | | Emergency Nurse Essentials | | Irrelevant | | | | Irrelevant | | | | Irrelevant | |
| 12 | | e-Sonic Urgent Care | | Irrelevant | | | | Irrelevant | | | | Irrelevant | |
| 13 | | Heart Doctor ER Hospital Manager: Hospital Games | | Irrelevant | | | | Irrelevant | | | | Irrelevant | |
| 14 | | Dr. Cubas MIA Urgent Care | | Irrelevant | | | | Irrelevant | | | | Irrelevant | |
| 15 | | MediCode: AHA ACLS, BLS & PALS | | Irrelevant | | | | Irrelevant | | | | Irrelevant | |
| 16 | | Operate Now: Hospital | | Irrelevant | | | | Irrelevant | | | | Irrelevant | |
| 17 | | Alberta Health Services (AHS) | | Irrelevant | | | | Irrelevant | | | | Irrelevant | |
| 18 | | tigma | | Irrelevant | | | | Irrelevant | | | | Irrelevant | |
| 19 | | Emergency Nursing Pro | | Irrelevant | | | | Irrelevant | | | | Irrelevant | |
| 20 | | My Dream Hospital Doctor Games: Emergency Room | | Irrelevant | | | | Irrelevant | | | | Irrelevant | |
| **9 Enable people living with long-term conditions to improve their health**  **Search term: Long-term care** | | | | | | | | | | | | | |
| **No** | | **App** | | **AW**  **(Relevant or Irrelevant)** | | | | **SW**  **(Relevant or Irrelevant)** | | | | **Consensus** | |
| 1 | | Elder Pilot: Long-Term Care | | Irrelevant | | | | Irrelevant | | | | Irrelevant | |
| 2 | | MO Health Care Association | | Irrelevant | | | | Irrelevant | | | | Irrelevant | |
| 3 | | Managed Health Care Connect | | Irrelevant | | | | Irrelevant | | | | Irrelevant | |
| 4 | | Zintelis | | Irrelevant | | | | Irrelevant | | | | Irrelevant | |
| 5 | | NH Regulations | | Irrelevant | | | | Irrelevant | | | | Irrelevant | |
| 6 | | LTCUnderwriter | | Irrelevant | | | | Irrelevant | | | | Irrelevant | |
| 7 | | JAMDA | | Irrelevant | | | | Irrelevant | | | | Irrelevant | |
| 8 | | easyDOK mobile *(non-English)* | | Irrelevant | | | | Irrelevant | | | | Irrelevant | |
| 9 | | GoToStock ( INTRADAY SHORT LONG TERM BTST PICKS ) | | Irrelevant | | | | Irrelevant | | | | Irrelevant | |
| 10 | | ChemRx | | Irrelevant | | | | Irrelevant | | | | Irrelevant | |
| 11 | | Gerontology | | Irrelevant | | | | Irrelevant | | | | Irrelevant | |
| 12 | | ASG | | Irrelevant | | | | Irrelevant | | | | Irrelevant | |
| 13 | | Expert Stewardship, Inc. | | Irrelevant | | | | Irrelevant | | | | Irrelevant | |
| 14 | | Hourly weather forecast and long-term report | | Irrelevant | | | | Irrelevant | | | | Irrelevant | |
| 15 | | Nursing Info | | Irrelevant | | | | Irrelevant | | | | Irrelevant | |
| 16 | | Remente - Self Improvement | | Irrelevant | | | | Irrelevant | | | | Irrelevant | |
| 17 | | Dietitian's Toolbox | | Irrelevant | | | | Irrelevant | | | | Irrelevant | |
| 18 | | Goal Planner: Habit Tracker & Goal Setting Manager | | Irrelevant | | | | Irrelevant | | | | Irrelevant | |
| 19 | | My Eyes Health Protection App | | Irrelevant | | | | Irrelevant | | | | Irrelevant | |
| 20 | | Fasting Time - Fasting Tracker & Weight Loss Clock | | Irrelevant | | | | Irrelevant | | | | Irrelevant | |
| **10** **Manage concurrent health problems in an individual patient**  **Search term: Health problems** | | | | | | | | | | | | | |
| **No** | | **App** | | **AW**  **(Relevant or Irrelevant)** | | | **SW**  **(Relevant or Irrelevant)** | | | | | **Consensus** | |
| 1 | | Ada - Your Health Guide | | Irrelevant | | | Irrelevant | | | | | Irrelevant | |
| 2 | | 🇬🇧Diagnosis Medical App | | Irrelevant | | | Irrelevant | | | | | Irrelevant | |
| 3 | | Health and Nutrition Guide | | Irrelevant | | | Irrelevant | | | | | Irrelevant | |
| 4 | | Total Health Care | | Irrelevant | | | Irrelevant | | | | | Irrelevant | |
| 5 | | Your.MD: Symptom Checker & Health Chatbot | | Irrelevant | | | Irrelevant | | | | | Irrelevant | |
| 6 | | Dictionary Diseases&Disorders | | Irrelevant | | | Irrelevant | | | | | Irrelevant | |
| 7 | | 101 Natural Home Remedies Cure | | Irrelevant | | | Irrelevant | | | | | Irrelevant | |
| 8 | | Daily Health & Fitness Tips | | Irrelevant | | | Irrelevant | | | | | Irrelevant | |
| 9 | | Gut Health Problems | | Irrelevant | | | Irrelevant | | | | | Irrelevant | |
| 10 | | My Health History | | Irrelevant | | | Irrelevant | | | | | Irrelevant | |
| 11 | | Home Remedies - Natural Cure | | Irrelevant | | | Irrelevant | | | | | Irrelevant | |
| 12 | | Bone and joint : diseases and treatments | | Irrelevant | | | Irrelevant | | | | | Irrelevant | |
| 13 | | All Skin Diseases and Treatment- A to Z | | Irrelevant | | | Irrelevant | | | | | Irrelevant | |
| 14 | | Top Liver Cleansing Superfoods | | Irrelevant | | | Irrelevant | | | | | Irrelevant | |
| 15 | | Animals Diseases And Cure 2018 | | Irrelevant | | | Irrelevant | | | | | Irrelevant | |
| 16 | | Common Health Problems (Wiki Kids Limited) | | Irrelevant | | | Irrelevant | | | | | Irrelevant | |
| 17 | | Health & Fitness Tracker with Calorie Counter | | Irrelevant | | | Irrelevant | | | | | Irrelevant | |
| 18 | | Fabulous: Motivate Me! Meditate, Relax, Sleep | | Irrelevant | | | Irrelevant | | | | | Irrelevant | |
| 19 | | Medicalog for Families | | Irrelevant | | | Irrelevant | | | | | Irrelevant | |
| 20 | | Common Health Problems (The Funny Grandpa) | | Irrelevant | | | Irrelevant | | | | | Irrelevant | |
| **11 Coordinate a team-based approach to the care of patients**  **Search term: Team-based care** | | | | | | | | | | | | | |
| **No** | **App** | | **AW**  **(Relevant or Irrelevant)** | | | | | | | **SW**  **(Relevant or Irrelevant)** | | | **Consensus** |
| 1 | AG PCNP Adult Primary Care MCQ Exam PRO | | Irrelevant | | | | | | | Irrelevant | | | Irrelevant |
| 2 | Dream Hospital - Health Care Manager Simulator | | Irrelevant | | | | | | | Irrelevant | | | Irrelevant |
| 3 | AG PCNP Adult Primary Care Exam Pre Quiz 2018 Ed | | Irrelevant | | | | | | | Irrelevant | | | Irrelevant |
| 4 | Patient Care & Health Safety Flashcards 2018 | | Irrelevant | | | | | | | Irrelevant | | | Irrelevant |
| 5 | Virtual Practice for Healthcare Providers | | Irrelevant | | | | | | | Irrelevant | | | Irrelevant |
| 6 | NCCN Guidelines for Smartphone | | Irrelevant | | | | | | | Irrelevant | | | Irrelevant |
| 7 | DIYA HEALTH CARE | | Irrelevant | | | | | | | Irrelevant | | | Irrelevant |
| 8 | Washington Township Medical | | Irrelevant | | | | | | | Irrelevant | | | Irrelevant |
| 9 | Home Health Care | | Irrelevant | | | | | | | Irrelevant | | | Irrelevant |
| 10 | HubChart™ | | Irrelevant | | | | | | | Irrelevant | | | Irrelevant |
| 11 | Medocity MD: Health Care Management | | Irrelevant | | | | | | | Irrelevant | | | Irrelevant |
| 12 | eJournal | | Irrelevant | | | | | | | Irrelevant | | | Irrelevant |
| 13 | NCCN Patient Guides for Cancer | | Irrelevant | | | | | | | Irrelevant | | | Irrelevant |
| 14 | CardioHFDoc | | Irrelevant | | | | | | | Irrelevant | | | Irrelevant |
| 15 | Community Eye Care | | Irrelevant | | | | | | | Irrelevant | | | Irrelevant |
| 16 | MILLENSYS Health Wallet | | Irrelevant | | | | | | | Irrelevant | | | Irrelevant |
| 17 | Minerva for patients | | Irrelevant | | | | | | | Irrelevant | | | Irrelevant |
| 18 | NICE Guidance | | Irrelevant | | | | | | | Irrelevant | | | Irrelevant |
| 19 | iUGO Care | | Irrelevant | | | | | | | Irrelevant | | | Irrelevant |
| 20 | Hero Hunters | | Irrelevant | | | | | | | Irrelevant | | | Irrelevant |
| **12 Support people through individual experiences of health, illness and recovery**  **Search term: Health promotion** | | | | | | | | | | | | | |
| **No** | **App** | | | **AW**  **(Relevant or Irrelevant)** | | | | | **SW**  **(Relevant or Irrelevant)** | | | **Consensus** | |
| 1 | Healthy 365 | | | Relevant | | | | | Relevant | | | Relevant | |
| 2 | HealthHub SG | | | Irrelevant | | | | | Irrelevant | | | Irrelevant | |
| 3 | Public Health Programs | | | Irrelevant | | | | | Relevant | | | Irrelevant | |
| 4 | HealthHub Track | | | Relevant | | | | | Relevant | | | Relevant | |
| 5 | IUHPE 2016 | | | Irrelevant | | | | | Irrelevant | | | Irrelevant | |
| 6 | A&SHPC 2016 | | | Irrelevant | | | | | Irrelevant | | | Irrelevant | |
| 7 | Oxford Medical Dictionary | | | Irrelevant | | | | | Irrelevant | | | Irrelevant | |
| 8 | Food(lg) | | | Relevant | | | | | Relevant | | | Relevant | |
| 9 | Public health | | | Irrelevant | | | | | Irrelevant | | | Irrelevant | |
| 10 | Melbourne Health History Walks | | | Irrelevant | | | | | Irrelevant | | | Irrelevant | |
| 11 | Health Talk Kit - 2018 Edition | | | Irrelevant | | | | | Irrelevant | | | Irrelevant | |
| 12 | Appibuddy | | | Relevant | | | | | Relevant | | | Relevant | |
| 13 | VicHealth | | | Irrelevant | | | | | Irrelevant | | | Irrelevant | |
| 14 | MBBS Study App | | | Irrelevant | | | | | Irrelevant | | | Irrelevant | |
| 15 | Health Nutrition and food Guide | | | Irrelevant | | | | | Irrelevant | | | Irrelevant | |
| 16 | TASHePA | | | Irrelevant | | | | | Irrelevant | | | Irrelevant | |
| 17 | Nursing Diagnosis Flashcards 2018 | | | Irrelevant | | | | | Irrelevant | | | Irrelevant | |
| 18 | Health Ideas | | | Irrelevant | | | | | Irrelevant | | | Irrelevant | |
| 19 | Sexual Health | | | Irrelevant | | | | | Irrelevant | | | Irrelevant | |
| 20 | OnDoctor | | | Irrelevant | | | | | Irrelevant | | | Irrelevant | |

**iOS apps (Team 2)**

| **1 Apply a structured approach to data gathering and investigation**  **Search term: History taking** | | | | | | | | |
| --- | --- | --- | --- | --- | --- | --- | --- | --- |
| **No** | **App** | | **CHT**  **(Relevant or Irrelevant)** | **WJW**  **(Relevant or Irrelevant)** | | | | **Consensus** |
| 1 | Taking a Walk through the street | | Irrelevant | Irrelevant | | | | Irrelevant |
| 2 | Pill Reminder Alarm- Reminder to Take Medicine | | Irrelevant | Irrelevant | | | | Irrelevant |
| 3 | Historian | | Relevant | Relevant | | | | Relevant |
| 4 | Hx Medical History Taking | | Irrelevant | Irrelevant | | | | Irrelevant |
| **2 Interpret findings accurately to reach a diagnosis**  **Search term: Diagnosis** | | | | | | | | |
| **No** | **App** | | **CHT**  **(Relevant or Irrelevant)** | **WJW**  **(Relevant or Irrelevant)** | | | **Consensus** | |
| 1 | Prognosis: Your Diagnosis | | Irrelevant | Irrelevant | | | Irrelevant | |
| 2 | Calculate by QxMD | | Irrelevant | Irrelevant | | | Irrelevant | |
| 3 | Emergency medicine: diagnosis & management | | Irrelevant | Irrelevant | | | Irrelevant | |
| 4 | Baby gender diagnosis | | Irrelevant | Irrelevant | | | Irrelevant | |
| 5 | DSM-5^TM^ differential diagnosis | | Irrelevant | Irrelevant | | | Irrelevant | |
| 6 | Differential diagnosis guide | | Irrelevant | Irrelevant | | | Irrelevant | |
| 7 | visualDx | | Irrelevant | Irrelevant | | | Irrelevant | |
| 8 | Diagnosis& therapy: the pro symptom checker & tracker for physical, occupational, Speech ddx & Blood Test Guide FREE! | | Irrelevant | Irrelevant | | | Irrelevant | |
| 9 | Clinical sense | | Irrelevant | Irrelevant | | | Irrelevant | |
| 10 | Your rapid diagnosis- STD | | Relevant | Relevant | | | Relevant | |
| 11 | X-ray differential diagnosis | | Irrelevant | Irrelevant | | | Irrelevant | |
| 12 | Common differential diagnosis | | Irrelevant | Irrelevant | | | Irrelevant | |
| 13 | MDCalc Medical Calculator | | Irrelevant | Irrelevant | | | Irrelevant | |
| 14 | TBI prognosis | | Irrelevant | Irrelevant | | | Irrelevant | |
| 15 | Nurse’s pocket guide- diagnosis | | Irrelevant | Irrelevant | | | Irrelevant | |
| 16 | Rapid diagnosis- mental health | | Relevant | Relevant | | | Relevant | |
| 17 | MIMS Malaysia | | Irrelevant | Irrelevant | | | Irrelevant | |
| 18 | BMJ best practice | | Irrelevant | Irrelevant | | | Irrelevant | |
| 19 | Intragen Institute- diagnosis and treatment of hair problems | | Irrelevant | Irrelevant | | | Irrelevant | |
| 20 | Voice Aura- Sound diagnosis-what color is your voice? | | Irrelevant | Irrelevant | | | Irrelevant | |
| **3 Demonstrate a proficient approach to clinical examination**  **Search term: Clinical examination** | | | | | | | | |
| **No** | **App** | | **CHT**  **(Relevant or Irrelevant)** | **WJW**  **(Relevant or Irrelevant)** | | | | **Consensus** |
| 1 | Clinical examination and Skills Free | | Irrelevant | Irrelevant | | | | Irrelevant |
| 2 | Clinical knowledge exam questions 2017 | | Irrelevant | Irrelevant | | | | Irrelevant |
| 3 | Clinical bundle : LabGear, Clinical examination& cranial nerve | | Irrelevant | Irrelevant | | | | Irrelevant |
| 4 | Clinical cases for GP exams | | Irrelevant | Irrelevant | | | | Irrelevant |
| 5 | Clinical Exam | | Irrelevant | Irrelevant | | | | Irrelevant |
| 6 | Geeky Medics- OSCE revision | | Irrelevant | Irrelevant | | | | Irrelevant |
| 7 | OSCE revision for medical students | | Irrelevant | Irrelevant | | | | Irrelevant |
| 8 | OSCE crash course: Clinical examination and skills | | Irrelevant | Irrelevant | | | | Irrelevant |
| 9 | DeGowin’s Diagnostic Examination Flashcards | | Irrelevant | Irrelevant | | | | Irrelevant |
| 10 | Clinical Anesthesia Exam | | Irrelevant | Irrelevant | | | | Irrelevant |
| 11 | UMSLE Clinical anatomy quiz | | Irrelevant | Irrelevant | | | | Irrelevant |
| 12 | Shelf Exam: internal medicine | | Irrelevant | Irrelevant | | | | Irrelevant |
| 13 | SnapDx Clinical- Evidence based Physical exam and Bedside Assessments | | Irrelevant | Irrelevant | | | | Irrelevant |
| 14 | CORE- Clinical Orthopedic Exam | | Irrelevant | Irrelevant | | | | Irrelevant |
| 15 | OH Clinical Exam& Paract. Skills | | Irrelevant | Irrelevant | | | | Irrelevant |
| 16 | Clinical skills- History, Symptoms& Physical examination | | Irrelevant | Irrelevant | | | | Irrelevant |
| 17 | Medical student bundle- Lab reference, renote for note taking, clinical skills, cranial nerve | | Irrelevant | Irrelevant | | | | Irrelevant |
| 18 | Clinical Anesthesia Exam Pro | | Irrelevant | Irrelevant | | | | Irrelevant |
| 19 | Practice UKCAT Questions | | Irrelevant | Irrelevant | | | | Irrelevant |
| 20 | Shelf Exam: Surgery | | Irrelevant | Irrelevant | | | | Irrelevant |
| **4 Demonstrate a proficient approach to the performance of procedures**  **Search term: Medical procedures** | | | | | | | | |
| **No** | **App** | | **CHT**  **(Relevant or Irrelevant)** | **WJW**  **(Relevant or Irrelevant)** | | | | **Consensus** |
| 1 | Medical and Surgical Procedures Free | | Irrelevant | Irrelevant | | | | Irrelevant |
| 2 | EM Logbook | | Irrelevant | Irrelevant | | | | Irrelevant |
| 3 | Medical and Surgical Procedures Full | | Irrelevant | Irrelevant | | | | Irrelevant |
| 4 | Practical Medical Procedures at a glance (pay) | | Irrelevant | Irrelevant | | | | Irrelevant |
| 5 | PCMED- CanMEDS and Case Log | | Irrelevant | Irrelevant | | | | Irrelevant |
| 6 | Anesthesia Procedures | | Irrelevant | Irrelevant | | | | Irrelevant |
| 7 | Lippincott Procedures | | Irrelevant | Irrelevant | | | | Irrelevant |
| 8 | Med Procedures- A La Carte | | Irrelevant | Irrelevant | | | | Irrelevant |
| 9 | Your Medical Encyclopedia | | Irrelevant | Irrelevant | | | | Irrelevant |
| 10 | Procedures in O&G | | Irrelevant | Irrelevant | | | | Irrelevant |
| 11 | RealWorld Procedures | | Irrelevant | Irrelevant | | | | Irrelevant |
| 12 | Anesthesia Procedures Ad free | | Irrelevant | Irrelevant | | | | Irrelevant |
| 13 | Lippincott Nursing Procedures | | Irrelevant | Irrelevant | | | | Irrelevant |
| 14 | Derm and Cosmetic Procedures | | Irrelevant | Irrelevant | | | | Irrelevant |
| 15 | CTCase Log | | Irrelevant | Irrelevant | | | | Irrelevant |
| 16 | Pfenninger& Fowler Procedures (pay) | | Irrelevant | Irrelevant | | | | Irrelevant |
| 17 | Family Medical Manager | | Irrelevant | Irrelevant | | | | Irrelevant |
| 18 | MyProcedures | | Irrelevant | Irrelevant | | | | Irrelevant |
| 19 | Clinical Anaesthesia Procedures | | Irrelevant | Irrelevant | | | | Irrelevant |
| 20 | Nursing Procedures | | Irrelevant | Irrelevant | | | | Irrelevant |
| **5 Adopt appropriate decision-making principles**  **Search term: Medical decision making** | | | | | | | | |
| **No** | **App** | | **CHT**  **(Relevant or Irrelevant)** | **WJW**  **(Relevant or Irrelevant)** | | | | **Consensus** |
| 1 | Medical Calculators Algorithms | | Irrelevant | Irrelevant | | | | Irrelevant |
| 2 | Medrills: Reassessment and Decision Making (pay) | | Irrelevant | Irrelevant | | | | Irrelevant |
| 3 | Decision Helper: make a choice a void bad decisions | | Irrelevant | Irrelevant | | | | Irrelevant |
| 4 | Billup (pay) | | Irrelevant | Irrelevant | | | | Irrelevant |
| 5 | Aspirin Guide | | Irrelevant | Irrelevant | | | | Irrelevant |
| 6 | SMDM | | Irrelevant | Irrelevant | | | | Irrelevant |
| 7 | NHS Pre-operative Test Checker | | Irrelevant | Irrelevant | | | | Irrelevant |
| 8 | ~~eBooks by lnkling~~ | | Irrelevant | Irrelevant | | | | Irrelevant |
| 9 | Bariatric Surgery Calculator | | Irrelevant | Irrelevant | | | | Irrelevant |
| 10 | sourceMD | | Irrelevant | Irrelevant | | | | Irrelevant |
| 11 | Status/post EDMS | | Irrelevant | Irrelevant | | | | Irrelevant |
| 12 | Darmkrebs CheckApp Shared Decision Making | | Irrelevant | Irrelevant | | | | Irrelevant |
| 13 | CIRSpotlight | | Irrelevant | Irrelevant | | | | Irrelevant |
| **6** **Adopt a structured approach to clinical management**  **Search term: Clinical management** | | | | | | | | |
| **No** | **App** | | **CHT**  **(Relevant or Irrelevant)** | **WJW**  **(Relevant or Irrelevant)** | | | | **Consensus** |
| 1 | MIHealth Forum- Health management & Clinical innovation | | Irrelevant | Irrelevant | | | | Irrelevant |
| 2 | Astracore Clinical | | Irrelevant | Irrelevant | | | | Irrelevant |
| 3 | Alcoholism: clinical and experimental research | | Irrelevant | Irrelevant | | | | Irrelevant |
| 4 | AfA HIV clinical guidelines | | Irrelevant | Irrelevant | | | | Irrelevant |
| 5 | Breastfeeding management 2 (pay) | | Irrelevant | Irrelevant | | | | Irrelevant |
| 6 | Management of candidemia in Stewardship Era | | Irrelevant | Irrelevant | | | | Irrelevant |
| 7 | Non small cell lung cancer | | Irrelevant | Irrelevant | | | | Irrelevant |
| 8 | Aetasapp | | Irrelevant | Irrelevant | | | | Irrelevant |
| 9 | DPCG postoperative management | | Irrelevant | Irrelevant | | | | Irrelevant |
| 10 | Healogics Document management | | Irrelevant | Irrelevant | | | | Irrelevant |
| 11 | CNL: clinical nurse leader Q&A | | Irrelevant | Irrelevant | | | | Irrelevant |
| 12 | Nursing leadership& management Exam Review (pay) | | Irrelevant | Irrelevant | | | | Irrelevant |
| 13 | DrChrono HER/EMR | | Irrelevant | Irrelevant | | | | Irrelevant |
| 14 | The Health care Provider’s guide to breastfeeding | | Irrelevant | Irrelevant | | | | Irrelevant |
| 15 | The springer healthcare oncology library | | Irrelevant | Irrelevant | | | | Irrelevant |
| 16 | insight CR | | Irrelevant | Irrelevant | | | | Irrelevant |
| 17 | Lab medicine digital | | Irrelevant | Irrelevant | | | | Irrelevant |
| 18 | STARLIMS | | Irrelevant | Irrelevant | | | | Irrelevant |
| 19 | RadsBest | | Irrelevant | Irrelevant | | | | Irrelevant |
| 20 | iGrade Social Worker | | Irrelevant | Irrelevant | | | | Irrelevant |
| **7 Make appropriate use of other professionals and services**  **Search term: Health professionals** | | | | | | | | |
| **No** | **App** | | **CHT**  **(Relevant or Irrelevant)** | **WJW**  **(Relevant or Irrelevant)** | | | | **Consensus** |
| 1 | Mobile Productivity for health professionals | | Irrelevant | Irrelevant | | | | Irrelevant |
| 2 | Private health professionals | | Irrelevant | Irrelevant | | | | Irrelevant |
| 3 | D2DUC for health professionals | | Irrelevant | Irrelevant | | | | Irrelevant |
| 4 | Mobile health library | | Irrelevant | Irrelevant | | | | Irrelevant |
| 5 | Hair professionals salon | | Irrelevant | Irrelevant | | | | Irrelevant |
| 6 | Depression test | | Irrelevant | Irrelevant | | | | Irrelevant |
| 7 | Psych terms: Medical dictionary and terminology pocket glossary for psychology, psychiatry & mental health | | Irrelevant | Irrelevant | | | | Irrelevant |
| 8 | Your child’s first health record (bluebook) | | Irrelevant | Irrelevant | | | | Irrelevant |
| 9 | Professionals epilepsy manual | | Irrelevant | Irrelevant | | | | Irrelevant |
| 10 | Psych drugs & medications: psychiatric meds guide | | Irrelevant | Irrelevant | | | | Irrelevant |
| 11 | Yoga alliance professionals | | Irrelevant | Irrelevant | | | | Irrelevant |
| 12 | Karify for professionals | | Irrelevant | Irrelevant | | | | Irrelevant |
| 13 | Totallook style professionals | | Irrelevant | Irrelevant | | | | Irrelevant |
| 14 | Drug pronunciations Lite | | Irrelevant | Irrelevant | | | | Irrelevant |
| 15 | App for Avigo professionals | | Irrelevant | Irrelevant | | | | Irrelevant |
| 16 | Salus- Book fitness & wellness professionals near you | | Irrelevant | Irrelevant | | | | Irrelevant |
| 17 | Latest health news | | Irrelevant | Irrelevant | | | | Irrelevant |
| 18 | Ciao bella- Hair& beauty professionals | | Irrelevant | Irrelevant | | | | Irrelevant |
| 19 | Server health checker | | Irrelevant | Irrelevant | | | | Irrelevant |
| 20 | iCU notes | | Irrelevant | Irrelevant | | | | Irrelevant |
| **8 Provide urgent care when needed**  **Search term: Urgent care** | | | | | | | | |
| **No** | **App** | | **CHT**  **(Relevant or Irrelevant)** | **WJW**  **(Relevant or Irrelevant)** | | | | **Consensus** |
| 1 | Urgent care RAP | | Irrelevant | Irrelevant | | | | Irrelevant |
| 2 | XpediCare Online urgent care | | Irrelevant | Irrelevant | | | | Irrelevant |
| 3 | MASH urgent care | | Irrelevant | Irrelevant | | | | Irrelevant |
| 4 | Find urgent care centers | | Irrelevant | Irrelevant | | | | Irrelevant |
| 5 | Get med urgent care | | Irrelevant | Irrelevant | | | | Irrelevant |
| 6 | Soins urgent care | | Irrelevant | Irrelevant | | | | Irrelevant |
| 7 | Doctor on demand | | Irrelevant | Irrelevant | | | | Irrelevant |
| 8 | ExpressCare urgent care | | Irrelevant | Irrelevant | | | | Irrelevant |
| 9 | Tanner urgent care | | Irrelevant | Irrelevant | | | | Irrelevant |
| 10 | APLUS urgent care | | Irrelevant | Irrelevant | | | | Irrelevant |
| 11 | Healthy urgent care | | Irrelevant | Irrelevant | | | | Irrelevant |
| 12 | Urgent care PHR | | Irrelevant | Irrelevant | | | | Irrelevant |
| 13 | WVU urgent care | | Irrelevant | Irrelevant | | | | Irrelevant |
| 14 | Garrison Family-AV urgent care | | Irrelevant | Irrelevant | | | | Irrelevant |
| 15 | Central jersey urgent care | | Irrelevant | Irrelevant | | | | Irrelevant |
| 16 | Pocketdoc- urgent care | | Irrelevant | Irrelevant | | | | Irrelevant |
| 17 | MIA urgent care | | Irrelevant | Irrelevant | | | | Irrelevant |
| 18 | Immediate urgent care | | Irrelevant | Irrelevant | | | | Irrelevant |
| 19 | MAP-health urgent care and hospital doctor | | Irrelevant | Irrelevant | | | | Irrelevant |
| 20 | Xpress wellness urgent care | | Irrelevant | Irrelevant | | | | Irrelevant |
| **9 Enable people living with long-term conditions to improve their health**  **Search term: Long-term care** | | | | | | | | |
| **No** | **App** | | **CHT**  **(Relevant or Irrelevant)** | **WJW**  **(Relevant or Irrelevant)** | | | | **Consensus** |
| 1 | SpecialtyRx now | | Irrelevant | Irrelevant | | | | Irrelevant |
| 2 | Managed health care connect | | Irrelevant | Irrelevant | | | | Irrelevant |
| 3 | Day care-family | | Irrelevant | Irrelevant | | | | Irrelevant |
| 4 | CNA study guide | | Irrelevant | Irrelevant | | | | Irrelevant |
| 5 | DBS FASTerm | | Irrelevant | Irrelevant | | | | Irrelevant |
| 6 | Nursing home administration | | Irrelevant | Irrelevant | | | | Irrelevant |
| 7 | MNU app | | Irrelevant | Irrelevant | | | | Irrelevant |
| 8 | TransQuote mobile | | Irrelevant | Irrelevant | | | | Irrelevant |
| 9 | Mediprocity | | Irrelevant | Irrelevant | | | | Irrelevant |
| 10 | CareAscend | | Irrelevant | Irrelevant | | | | Irrelevant |
| 11 | Odona app | | Irrelevant | Irrelevant | | | | Irrelevant |
| 12 | MedTablet ADL | | Irrelevant | Irrelevant | | | | Irrelevant |
| 13 | My ALF training | | Irrelevant | Irrelevant | | | | Irrelevant |
| 14 | ASAP chat | | Irrelevant | Irrelevant | | | | Irrelevant |
| 15 | ADL calc | | Irrelevant | Irrelevant | | | | Irrelevant |
| 16 | eCLTC | | Irrelevant | Irrelevant | | | | Irrelevant |
| 17 | MDS assistant | | Irrelevant | Irrelevant | | | | Irrelevant |
| 18 | Goldcare Mobility Plus | | Irrelevant | Irrelevant | | | | Irrelevant |
| 19 | Activ8rlives^4^ Health+ Wellness | | Irrelevant | Irrelevant | | | | Irrelevant |
| 20 | Zintelis | | Irrelevant | Irrelevant | | | | Irrelevant |
| **10** **Manage concurrent health problems in an individual patient**  **Search term: Health problems** | | | | | | | | |
| **No** | **App** | | **CHT**  **(Relevant or Irrelevant)** | **WJW**  **(Relevant or Irrelevant)** | | | | **Consensus** |
| 1 | Cat’s Health problems +Info (pay) | | Irrelevant | Irrelevant | | | | Irrelevant |
| 2 | Gut Health problems | | Irrelevant | Irrelevant | | | | Irrelevant |
| 3 | Common children health problems (pay) | | Irrelevant | Irrelevant | | | | Irrelevant |
| 4 | The vitamin cure for children’s health problem (pay) | | Irrelevant | Irrelevant | | | | Irrelevant |
| 5 | Skin disease problems and care | | Irrelevant | Irrelevant | | | | Irrelevant |
| 6 | Heart health- controlling high blood pressure and cholesterol to reduce cardiac risk factos | | Irrelevant | Irrelevant | | | | Irrelevant |
| 7 | Tibot- a trained eye | | Irrelevant | Irrelevant | | | | Irrelevant |
| 8 | Men’s sexual problems | | Irrelevant | Irrelevant | | | | Irrelevant |
| 9 | Home remedies for hair problems | | Irrelevant | Irrelevant | | | | Irrelevant |
| 10 | Sexual problems (pay) | | Irrelevant | Irrelevant | | | | Irrelevant |
| 11 | Veteran mental health | | Irrelevant | Irrelevant | | | | Irrelevant |
| 12 | Solve your acne problems (pay) | | Irrelevant | Irrelevant | | | | Irrelevant |
| 13 | Bloody marrie- girl problems stickers | | Irrelevant | Irrelevant | | | | Irrelevant |
| 14 | Goodnight- BabyBus | | Irrelevant | Irrelevant | | | | Irrelevant |
| 15 | Joining forces | | Irrelevant | Irrelevant | | | | Irrelevant |
| 16 | Treatment of acne problems (pay) | | Irrelevant | Irrelevant | | | | Irrelevant |
| 17 | Conquer acne problems today (pay) | | Irrelevant | Irrelevant | | | | Irrelevant |
| 18 | Asanas- health benefits | | Irrelevant | Irrelevant | | | | Irrelevant |
| 19 | Skin care 101-beauty and health guide with tutorial video (pay) | | Irrelevant | Irrelevant | | | | Irrelevant |
| 20 | Gas trouble in stomach acidity | | Irrelevant | Irrelevant | | | | Irrelevant |
| **11 Coordinate a team-based approach to the care of patients**  **Search term: Team-based care** | | | | | | | | |
| **No** | **App** | **CHT**  **(Relevant or Irrelevant)** | | | | **WJW**  **(Relevant or Irrelevant)** | | **Consensus** |
| **12 Support people through individual experiences of health, illness and recovery**  **Search term: Health promotion** | | | | | | | | |
| **No** | **App** | | **CHT**  **(Relevant or Irrelevant)** | | **WJW**  **(Relevant or Irrelevant)** | | | **Consensus** |
| 1 | Health adventures | | Irrelevant | | Irrelevant | | | Irrelevant |
| 2 | MY Watsons | | Irrelevant | | Irrelevant | | | Irrelevant |
| 3 | The circle of health | | Relevant | | Relevant | | | Relevant |
| 4 | hiSG- Health Insights SG | | Irrelevant | | Irrelevant | | | Irrelevant |
| 5 | Sasa | | Irrelevant | | Irrelevant | | | Irrelevant |
| 6 | Healthy 365 | | Relevant | | Relevant | | | Relevant |
| 7 | ProMotion Delta Faucet | | Irrelevant | | Irrelevant | | | Irrelevant |
| 8 | Melbourne Health History Walks | | Irrelevant | | Irrelevant | | | Irrelevant |
| 9 | HealthHub SG | | Irrelevant | | Irrelevant | | | Irrelevant |
| 10 | Vaniday | | Irrelevant | | Irrelevant | | | Irrelevant |
| 11 | NCLEX Practice Pro | | Irrelevant | | Irrelevant | | | Irrelevant |
| 12 | RiseToday: Yoga & Fitness | | Irrelevant | | Irrelevant | | | Irrelevant |
| 13 | USMLE@ Step 2 Exam Prep 2017 Edition | | Irrelevant | | Irrelevant | | | Irrelevant |
| 14 | Ga ned I vekt med hypnoterapi (pay) | | Irrelevant | | Irrelevant | | | Irrelevant |
| 15 | Lindre hodepine-hypoterapi (pay) | | Irrelevant | | Irrelevant | | | Irrelevant |
| 16 | MHT-COPD-Selfcare | | Irrelevant | | Irrelevant | | | Irrelevant |
| 17 | myChange | | Irrelevant | | Irrelevant | | | Irrelevant |
| 18 | Zmartsave | | Irrelevant | | Irrelevant | | | Irrelevant |
| 19 | HealthWatch | | Relevant | | Relevant | | | Relevant |
| 20 | Check My Food | | Irrelevant | | Irrelevant | | | Irrelevant |
